# The Association between Drinking Water Quality and Inflammatory Bowel Disease—A Study in Eastern Croatia

**DOI:** 10.3390/ijerph17228495

**Published:** 2020-11-16

**Authors:** Dubravka Holik, Atila Bezdan, Monika Marković, Želimir Orkić, Andrea Milostić-Srb, Štefica Mikšić, Aleksandar Včev

**Affiliations:** 1 Faculty of Dental Medicine and Health, Josip Juraj Strossmayer University of Osijek, 31000 Osijek, Croatia; dubravka.holik@fdmz.hr (D.H.); amsrb@fdmz.hr (A.M.-S.); stefica.miksic@fdmz.hr (Š.M.); aleksandar.vcev@fdmz.hr (A.V.); 2 Faculty of Agriculture, University of Novi Sad, 21000 Novi Sad, Serbia; atila.bezdan@polj.uns.ac.rs; 3 Faculty of Agrobiotechnical Sciences, Josip Juraj Strossmayer University of Osijek, 31000 Osijek, Croatia; 4 Faculty of Medicine, Josip Juraj Strossmayer University of Osijek, 31000 Osijek, Croatia; zelimir.orkic@gmail.com

**Keywords:** inflammatory bowel disease, ulcerative colitis, Crohn’s disease, Eastern Croatia, drinking water quality, rural public supply, private wells

## Abstract

The incidence rate of inflammatory bowel disease (IBD) is becoming a global health problem that could be caused by changes in environmental and lifestyle habits. The study aimed to identify the association between the quality of drinking water, i.e., physiochemical and biological aspects of the phenotype and activity of IBD in Eastern Croatia. The study included 312 patients (63.4% ulcerative colitis, UC, and 36.6% Crohn’s disease, CD) from the area of Eastern Croatia. The data were collected by questionnaires and the analysis of the water safety, based on 65 samples of drinking water by the patient’s water supply method (public supply, rural water supply, and private well). IBD was active in 38.0% patients (34.0% CD and 40.0% UC). Significant differences (*p* = 0.001) were observed in the distribution of patients, according to counties in which they lived in. The largest deviation was noted in coliform bacteria, *Escherichia coli*, and enterococci bacteria, Fe, Al, and nitrate in rural water supply and private wells, although, without significant impact on IBD phenotype and activity. The hazard quotient (HQ) simulations showed that children are a sensitive group, regarding exposure to nitrates in drinking water over a long period of time, so there is a need for further monitoring and analysis of this issue.

## 1. Introduction

Inflammatory bowel disease (IBD) is a term used for chronic gastrointestinal tract disorders, ulcerative colitis (UC), and Crohn’s disease (CD). IBD is specific for chronic inflammation and destruction of the intestinal wall, and the periods of exacerbation and remission of clinical symptoms [1,2,3]. Due to the unpredictable course, chronicity of the disease, and diagnosis at a young age, the need for expensive drugs and hospitalization for patients with IBD is a major public health problem. According to available data, it is estimated that more than three million people in Europe will contract IBD [4]. Several authors [5,6] have reported an increasing incidence and prevalence of IBD in all regions of the world, with considerable variations between countries. A high IBD incidence is noted in North America and northern and western Europe, while eastern Europe and Asian countries record an increase in newly affected patients [3,7].

As for Europe, Segerman et al. have stated that the Faroe Islands have the highest incidence of IBD in Europe [8]. Several authors [9,10] claim that the incidence of UC is twice that of CD in Asia as compared to the west. Studies previously published by Burisch et al. show a north–south gradient in more economically developed countries (MEDC), although with increasing incidences of IBD, the west–east gradient is emerging as well [11]. The incidence of IBD is almost twice as high in western Europe as in eastern Europe. It is interesting to note that, in eastern Europe, the UC to CD incidence ratio is 1:1, while in some countries, the incidence of CD is higher than UC, with Croatia as an example [12,13,14]. Some authors [15,16] claim that increased incidences may be caused by environmental and lifestyle factors. Furthermore, diseases are equally affecting men and women, with less emphasis on the more frequent occurrence of CD in women [5].

Experts agree that IBD etiology is multifactorial and that the significant factor of etiology is a complex interaction of genetic susceptibility, the immune system, the intestinal microbiome, and various environmental factors [3,17,18]. Fofanova et al. [2] presented a thorough analysis of microbiome–epigenome interactions and the environmental origins of IBD. The author claims that the genetic predisposition within children with very early-onset IBD appears to play a more important role, while environmental factors and gut microbiota are likely more involved in the disease etiology and natural history of patients (who present with the disease at a later age). Morgan et al. [19] pointed out that gastrointestinal microbiome composition, microbial function, and metabolic activity of gut microbiota are perturbed in patients suffering from IBD, and that is unclear whether this is an initiator or consequence of the disease. Actually, it is considered that environmental factors and lifestyle habits carry 70% of the risk occurrences of IBD, and that they have significant effects in the course of these diseases, although data are still limited [20,21]. It should be noted that, besides genetics, eating habits, and lifestyle, the quality and content of drinking water (organic and inorganic components) is considered as a risk factor for the development of IBD [22].

A Norwegian study aimed at examining the association between the presence of heavy metals and organic components in drinking water. The incidence rate of IBD confirmed the association of iron content in drinking water with an increased risk of developing IBD. Moreover, the association of the risk of IBD with pH and coliforms in drinking water was detected, although the effects were negligible [23].

According to Fathmawati et al., consuming high nitrates in drinking water can continuously harm human health. Usually, maximum nitrate concentration in public water supplies is rarely above the maximum contaminant level (MCL, 50 mg/L as nitrate or 11 mg/L as Nitrate-N) since it is more protected and controlled by regional water authorities [24,25]. This is opposite to agricultural regions, especially where the main source of drinking water is shallow wells. The main sources of nitrogen in drinking water are intensive agricultural production, meaning, nitrogen fertilizers in crop production, manure, and slurries from animal farms (diffuse source from agricultural run-off), but also point sources, such as septic tanks. It is important to emphasize that the MCL of nitrate is determined according to the occurrence of the methemoglobinemia, while the impact on the occurrence of other diseases has not been investigated.

Fathmawati et al. [24] stated that the nitrate pollution in water wells by human excreta can be correlated with the distance of wells to septic tanks (less than 10 m). Research conducted by Manbber et al. [22] aimed to investigate the relationship between quantity, type, and source of water, and the use of water purification devices with the onset rate of IBD. The primary outcome was the association between the amount of consumed water and IBD, and the secondary outcome was the impact of the amount, type, source of water, or use of water purification devices on the incidence of IBD. Research has shown that there is no significant correlation between the amount or source of water, and the presence or absence of IBD [22].

Despite the high prevalence and incidence of IBD in the south of Sweden, research aimed at drinking water analysis for pH, alkaline, nitrate, sulfate, iron, magnesium, and calcium compounds with the prevalence of IBD found no significant association [8]. Of course, other compounds in the drinking water could be connected to influence the risk. Bacterial contamination could reduce water quality. That is why the analysis of bacterial content should be carried out regularly, and if any signs of decontamination are present, the distribution should be stopped, and the population should be warned. Therefore, this mechanism is less likely to occur [8].

In Croatia, drinking water quality standards are under the responsibility of the Ministry of Health and Social Welfare. The Ministry is responsible for the monitoring of the sanitary quality of drinking water with the help of the Croatian National Institute of Public Health. The drinking water quality standards in Croatia, laid down by the ordinance on the sanitary quality of drinking water, are fully in line with the Directive’s 98/83/European Community (EC) and World Health Organization (WHO) requirements. In the period from 2016 to 2018, on average, across counties, more than 60% of collected water samples in Croatia were faulty, primarily because of the microbiological contaminants [26]. According to the European Commission, the most significant pressure in Croatia is on surface water bodies (57% of surface water bodies), while for groundwater bodies (6%), the most significant pressure is diffuse pollution from agricultural production [27]. As for the Nitrate Directive, Croatia designated approximately 9% of its territory as a nitrate-vulnerable zone (NVZ), which is not systematically associated with all areas subject to high agricultural pressure [28]. Croatia is one of the European countries with the lowest groundwater nitrate concentrations, generally below 20 mg NO_3_^−^/L, while in the southern, karst areas, they rarely exceed 5 mg/L [29]. It should be emphasized that the average annual input of nitrogen fertilizers in Croatia is considerably reduced (384 kg/ha in 2008, 180 kg/ha in 2016) because of the implementation of the Nitrate Directive [30]. Regardless, the average annual N and P fertilizer input in Croatia is below the European Union (EU) average [31].

Due to its complexity, it is clear that a multidisciplinary approach is necessary for basic and clinical IBD researches. During the last decade, significant IBD study advances have occurred with the development of cross-sectional studies, which include epidemiology, environmental factors, genetics, IBD diagnoses, medical therapy, etc. Cross-sectional studies are widely adopted in medical studies as a type of observational study that analyses data from a population at a specific point of time. Of course, it is important to indicate some limitations within, for example, inconsistent measurements of exposures to individual factors, such as diet, water quality, lifestyle habits, etc. Similar to many other countries, there is a lack of large population-based cohort studies of IBD in Croatia, due to the lack of clinical data or the difficulty in collecting sufficient patients in a long period of time.

For the current study, it is important to point out that no population-based study is currently being conducted in Croatia to determine the incidence and prevalence of IBD caused by the quality of drinking water. Therefore, the present study aims to identify the association between the quality of drinking water, i.e., physiochemical and biological aspects of the phenotype and activity of the IBD in Eastern Croatia.

## 2. Materials and Methods

### 2.1. Study Area

The study area is in the northern Pannonian region of Croatia where large lowlands are dominant. This area belongs to the continental climate region with cold winters, hot summers, and a mean annual precipitation of 600 mm. The study area consists of 3 counties, Požega-Slavonia (26.248 inhabitants, 133.9 km^2^), Virovitica-Podravina (74.521 inhabitants, 2.024 km^2^), and Vukovar-Srijem County (179.521 inhabitants, 2.454 km^2^), with a dominant agricultural sector due to fertile soils, where nitrate contamination in groundwater from fertilizer and animal manure could be an issue. Average input of N fertilizers in Virovitica-Podravina County is 101 kg/ha of mineral and 20 kg/ha of organic N, in Požega-Slavonia County, 97 kg/ha of mineral and 17 kg/ha of organic N, and in Vukovar-Srijem County, 110 kg/ha of mineral and 30 kg/ha of organic N [32], which is below the EU average [30].

### 2.2. Study Design and Participants

This cross-sectional study, according to Kogevinas et al. [33], was conducted from January to June 2016 to show the association between drinking water quality and the activities of CD and UC. In this study, only the IBD patients who developed specific conditions were included, i.e., not the entire population of the selected counties. The study contains individual-level data, meaning one record per patient (individual). Patients included in the study (312) were treated for IBD at Vukovar General County Hospital, Vinkovci General County Hospital, Požega General County Hospital, and Virovitica General County Hospital. The study was approved by the Ethics Committee of the Josip Juraj Strossmayer University of Osijek, Faculty of Medicine in Osijek (approval no. 2158-1-07-17-19).

Each patient was contacted to conduct a survey and to collect drinking water samples. The survey for this study was conducted with a questionnaire consisting of 42 questions divided into several categories: questions related to the sociodemographic characteristics of the patients; to personal and family history and disease activity (Harvey–Bradshaw index for assessment of Crohn’s disease activity, Mayo index for evaluation of UC activity, and disease phenotype according to the Montreal classification). The next category concerns respondents’ life habits and the questions related to exposure to environmental risk factors, i.e., water supply method and drinking water quality.

### 2.3. Water Analysis

Water sampling for biological and physiochemical analysis was performed by the following procedure. Before sampling, the field sampling was coordinated with the laboratory so that the water samples could get analyzed in the shortest period of time. For the analysis, glass sample bottles with a capacity of 500 mL were used. Since the water was chlorinated, sampling bottles were previously prepared in the laboratory, i.e., sodium thiosulfate was added to neutralize chlorine. Before sampling, the attachments from the tap were removed, and the tap was wiped so that the dirt was removed. Sampling bottles were labeled (date, patients name, location, water source) and taken with rubber gloves, so that contamination was avoided. At the patient locations, the cold water tap was turned on at maximum flow for 5 to 10 min. The bottles were filled carefully to prevent overfill and sealed with screw caps. Bottles with water samples were placed in a container and immediately taken to the laboratory.

In total, 65 drinking water samples were collected (sampling methods HRN ISO 5667-6: 2011; HRN EN ISO 19458: 2008) and submitted to the laboratory where the analysis was performed within six hours of sampling. Water samples were representative of locations at which water was delivered to the patient (and points of use). Drinking water samples were taken following the patient’s water supply method, i.e., tap water. One sample of water was sufficient for all patients from a certain location where the public water supply system or rural water supply system was the source of water. In cases where patients were supplied from private wells or tanks, drinking water samples were taken from each patient’s location separately.

Parameters of water quality were analyzed according to Ordinance on analytical methods (“Official Gazette”, No. 125/17), which is regulated by the law on the water intended for human consumption, provision of Council Directive (1998/83/EU) on the quality of water intended for human consumption, and European Commission Directive (EU) 2015/1787), the quality of water intended for human consumption.

The following water physicochemical parameters were analyzed: color (HRN EN ISO 7887: 2001 method), turbidity (HRN EN ISO 7027: 2012 method), odor and taste (Drinking water—standard methods for hygiene testing, 1990), pH value at 25 °C (HRN EN method ISO 10523: 2012), electrical conductivity (EC) at 25 °C (HRN method EN 27888: 2008), consumption KMnO4 (drinking water—Standard methods for hygiene testing, 1990), chlorides (HRN method ISO 9297: 1998), ammonia(method HRN ISO 7150-1: 1998), nitrates (Standard methods, 1975), nitrites (HRN EN 26777: 1998 method), iron (ASTM standard methods, 1981), aluminum (drinking water—standard methods for hygiene testing, 1990), manganese (Merck 1974/ASTM standard methods, 1981), arsenic (HRN EN ISO 15586: 2008 method). Total hardness analysis is expressed in the German unit (°dH, degree German hardness) and is the sum of the molar concentrations of Ca^2+^ and Mg^2+^.

The following microbiological analyses were performed: total coliforms (HRN EN ISO 9308-1: 2000 method), Escherichia coli (HRN EN ISO 9308-1: 2000 method), colonies at 37 °C/48h (HRN EN ISO 6222: 2000 method), colonies at 22 °C/72 h (HRN EN ISO 6222: 2000 method), enterococci (HRN EN ISO 7899-2: 2000).

Laboratory analyses of water samples were performed at the Health Ecology Service of the Brod-Posavina County accredited by the Croatian Accreditation Agency (HAA) according to the standard HRN EN ISO/IEC 17025: 2007.

### 2.4. Data Analysis

Categorical data is presented in absolute and relative frequencies. Numerical data is described using an arithmetic average and standard deviation for normal data distribution, and median and interquartile range for the remaining cases. The differences in categorical variables were tested using the χ^2^ test and Fisher’s exact test if necessary. The differences in normally distributed numerical variables between two independent groups were tested using the Student’s t-test, and in case of deviation from the normal distribution, the Mann–Whitney U test. All of the *p* values are two-tailed. The significance of differences (*p* < 0.05) determined by statistical testing is expressed at the level *p* < 0.05. The correlation analysis was used to test the strength of the relationship between nitrate concentration and the microbiological compounds in drinking water with the private well depth and the distance of livestock manure storage (Statistica 12, StatSoft, Tulsa, OK, USA).

The Montreal classification (modified Vienna system) was performed for subclassification of CD by phenotype, where the age of onset, disease location, and disease behavior as the predominant phenotypic elements were considered. The Mayo index (Mayo score), as the most commonly used index in clinical studies, was used for assessment of the severity of the UC, i.e., active and inactive disease. Disease activity is marked according to the sum of points as remission or inactive disease (0–1), mild disease (2–4), moderate disease (5–6), or severe disease (7–9). The Harvey–Bradshaw index (HBI) was used for assessing the degree of illness (activity) in patients with CD. Four disease categories are defined according to the sum of points: disease remission—inactive disease (sum of points 0–5), mild disease (sum of points 5–7), moderate disease (sum of points 8–16), and severe illness (sum of points greater than 16).

The Monte Carlo simulations were used in this study to perform the variability and sensitivity analysis of the risk assessment model predictions on the general population. Simulations were implemented using R and they were run for 10,000 iterations. The result of Monte Carlo simulations provide a confidence interval of the health risk of nitrate exposure from water consumption. Considering the variance of nitrate concentrations in water samples, this equation variable was defined in the terms of a probability density function taken from several measurements. A goodness of fit test was performed to select the most adequate distribution of nitrate concentrations. In this study, a health risk assessment of nitrate exposure in drinking water was carried out in three groups of the population, including children, adult females, and adult males. The risk assessment was performed based on water samples from public water supply (35 samples), but also groundwater wells (23 samples). Daily nitrate consumption was calculated as [34,35]:

(1) $$EDI=\frac{C_{f}\cdot C_{d}}{B_{w}}$$

where *EDI* is an estimation of daily nitrate consumption (mg/kg); *C_f_* is nitrate concentration in drinking water (mg/L), *C_d_* is average daily drinking water intake; and *B_w_* is body weight (kg). The World Health Organization [36] suggests that based on a 70 kg adult male, a 58 kg adult female, and a 10 kg child, under average conditions, it was estimated that adult males need 2.9 L of water per day, females needed 2.2 L/day and children 1.0 L/day. The health risk from water consumption was calculated as hazard quotient (HQ) [36,37]:

(2) $$HQ=\frac{EDI}{RFD}$$

The *RFD* represents the reference dose of nitrates, which is expressed in mg/kg body weight per day. According to the database of the Integrated Risk Information System guideline, the oral reference doses of nitrates are 1.6 mg/kg/day [37,38]. The value of *HQ* below 1 indicates that the harmful effects of exposure are not be expected, and values of *HQ* larger than 1 indicate that the health risks excess the acceptable level [35,39].

The examination of the correlation of certain factors to nitrate concentrations in private groundwater wells was performed. Chosen factors that may have had an impact on the nitrate concentrations included depth of wells, crop production, livestock, and pH of water. To determine the significance of each factor regarding its influence on nitrate concentration, the statistical analysis using logistic regression was used. Logistic regression was applied to predict a dependent binary response on nitrate concentration to independent variables [40]. An independent variable is considered significant if it has a *p*–value for Wald chi-square statistic less than 0.05 (95% confidence) [41]. The calculation of the logit coefficients was performed using R packages ISLR (New York, NY USA) [41] and Analysis of over-dispersed data (AOD) [42].

## 3. Results

From 312 patients (53.2% males and 46.8% females), 63.4% suffer from UC and 36.6% from CD. The phenotypic characteristics of the patients are presented in Table 1 and previously elaborated by Holik et al. [3].

The distribution of patients according to the county, length of the residence in the county, and settlement in urban or rural areas, are presented in Table 2. According to x^2^, analysis statistically significant differences (*p* = 0.001) is observed in the distribution of patients according to counties in which they are living, but without the significant impact of length of living. Moreover, there were no significant differences in patient distribution, according to the place of the residence.

### The Results of Water Analysis

**Figure 1:**
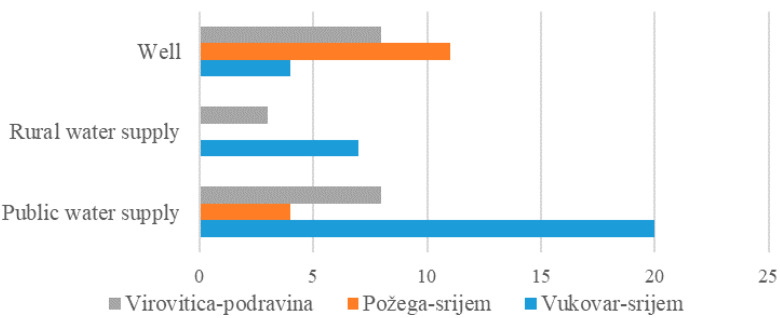
The number of water samples according to the type of water supply in the study area.

The number of water samples, according to the type of water supply in the study area, is presented in Figure 1. From a total of 65 drinking water samples, 31 samples were taken in Vukovar-Srijem, 15 samples from Požega-Slavonia, and 19 samples from Virovitica-Podravina county. 

**Figure 2:**
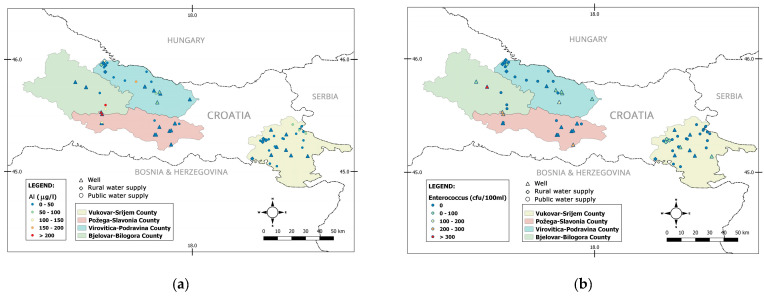

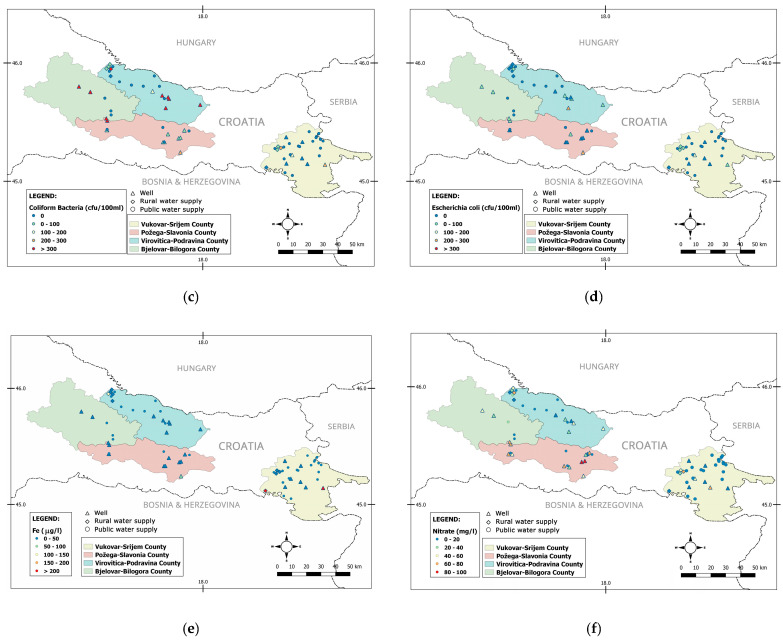
Water analysis; Al (**a**); Enterococcus (**b**); coliform bacteria (**c**); *Escherichia coli* (**d**); Fe (**e**); nitrate (**f**) by counties.

In general, the results of a biological and physicochemical analysis of drinking water differ among counties (Figure 2).

The analysis results of the physicochemical parameters of water samples are presented in Table 3. Values that exceed the MLC are highlighted in red. NO_3_^−^ ranged from 192 mg/L in Požega-Slavonia County to 0 mg/L in Virovitica-Podravina County. It is important to emphasize that all values that exceed MCL of NO_3_^−^ are found in private wells or rural water supply system.

In Požega-Slavonia County, all tested physiochemical parameters were within the allowable reference values.

In Virovitica-Podravina County, besides the NO_3_^−^ concertation, which was previously emphasized, the Al concentration was above MCL, 240 µg/L of Al was found in the public water supply system, while 730 µg/L in the private well (Table 3). The biggest concern in Vukovar-Srijem County was Fe concentrations, 712.2 µg/L of Fe was in the rural water supply system, and 223.5 µg/L of Fe in private wells. Followed by the high Fe concentration in rural water supply, there was a high Mn (184.8 µg/L) concentration in drinking water. As for As, the concentration that exceeded the MCL was found only in Vukovar-Srijem County. The maximum measured concentration was 213 µg/L, while the minimum measured concentration was 45 µg/L (Table 3).

In Požega-Slavonia County, total hardness ranged from 3.28 °dH in the public water supply to 43.54 °dH in a private well. In Virovitica-Podravina County, total hardness ranged from 4.39 °dH in the public water supply to 42.58 °dH in a private well. In Vukovar-Srijem County, total hardness ranged from 5.8 °dH in the public the water supply to 44.54 °dH in a private well. Overall, the highest total hardness is measured in private wells, and it is classified as very hard. In the public water supply, total hardness ranges from 3.28 °dH, i.e., very soft to 24.63 °dH or hard water.

The type of drinking water supply, as well as the water quality, did not significantly affect the IBD phenotype, as presented in Table 4.

**Figure 3:**
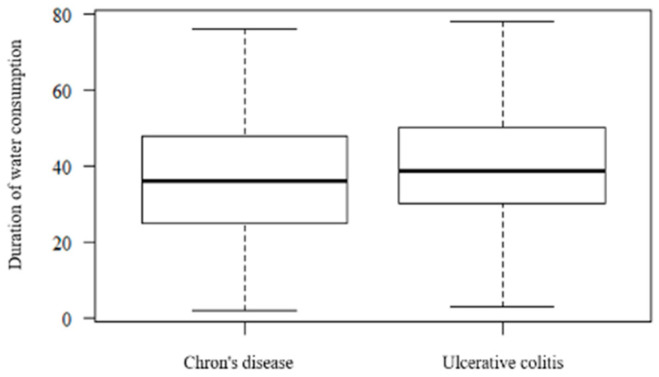
The patient’s distribution according to the duration of water consumption.

The duration of water consumption (Figure 3) did not significantly influence the IBD phenotype (Mann–Whitney U test, *p* = 0.132). Duration of water consumption refers to the period of time in which the patients consumed the tap water that was collected for the analysis.

The type of drinking water supply as well as the water quality did not significantly affect the IBD activity (Table 5).

**Figure 4:**
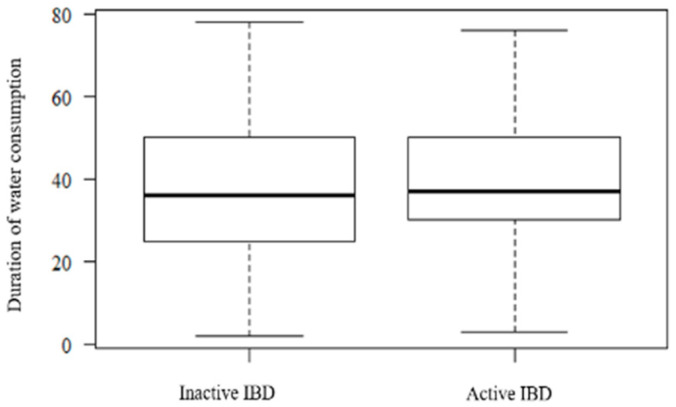
The patient’s distribution according to the duration of water consumption.

The period of water consumption (Figure 4) did not significantly influence the IBD activity (Mann–Whitney U test, *p* = 0.378).

The results of Monte Carlo simulations and hazard quotient (HQ) values with 90% confidence (P90) are presented in Table 6, for public water supply and private wells. The Monte Carlo simulation is usually used when there is the uncertainty of input parameter values or when inputs are subject to variability [43]. Using the R package “fitdistrplus” [44], the lognormal distribution is selected as most appropriate for representing nitrate concentrations in water samples from the public water supply and private wells.

The results of logistic regression are presented in Table 7. Results indicate that none of the chosen factors are significantly related (at 95% confidence) to nitrate concentration in well water.

## 4. Discussion

In Vukovar-Srijem County, 31 drinking water samples were analyzed; 20 (64.51%) from an urban public water supply, 7 (22.58%) from rural public water supply, and 4 (12.90%) from private wells. According to the study results, 29.03% of water samples were of poor quality. In Požega-Slavonia County, 15 water samples, of which 4 (26.67%) were from an urban public water supply and 11 (73.33%) from private wells. Almost 80.00% of water samples were of poor quality due to the biological pollutant. In Virovitica-Podravina County, 19 water samples were taken; 8 (42.10%) from an urban public water supply, 3 (15.79%) from rural water supply, and 8 (42.11%) from private wells, while 47.36% of water samples were of poor quality.

The largest deviation was noted in coliform bacteria, *Escherichia coli* enterococci bacteria, in rural water supply and private wells, although without a significant impact on IBD phenotype and activity. Here, we emphasize that the correlation analysis has shown a complete negative correlation between bacteria compounds and well depth (r= −0.99; *p* < 0.05; N = 50), more likely than the distance of livestock manure from the well (r = 0.1). This is partly contrary to the “hygiene hypothesis”, which states that individuals who are exposed to a healthy environment and high sanitary conditions are more likely at risk of IBD [45,46,47,48]. The study conducted in India [49] does not support the “hygiene hypothesis”, and also states that the higher incidence of UC could be associated with unhealthy sanitary conditions and other factors that should be identified in future studies. Particularly interesting studies are the ones that evaluated the industrialization and urbanization impact on IBD activity [12,17,50]. The mentioned studies confirmed that the expansion of industrialization and urbanization in comparison to life in rural areas has a considerably larger impact on IBD activity, meaning that higher living standards during childhood are connected with a higher risk of IBD. The NH_4_^+^, NO_3_^−^ concentrations above MCL are found in rural public supply and private wells in Vukovar-Srijem County, NO_3_^−^ concentrations above MCL in private wells in Požega-Slavonia and Virovitica-Podravina County.

The higher NO_3_^−^ concentration in private wells on family farms could be a result of shallow private wells (<20 m), more likely than the low distance of livestock manure storage. This is confirmed with correlation analysis; r = 0.6 for well depth and r = 0.45 for the distance of manure storage (*p* < 0.05; N = 50). Moreover, Fathmawati et al. [24] studied the impact of septic tank distance on nitrate concentration in waters from wells and found 92.6% of water samples that go beyond MCL in cases where the distance between the septic tank and well was less than 10 m.

In our study, Monte-Carlo simulation (MCS) is performed, since it is one of the most used approaches to stochastic modeling. The results of simulations of HQ values for public water supply with 90% confidence show that the levels of HQ of male and female adults are less than 1 (0.6 and 0.52, respectively), meaning that long-term exposure to nitrates in drinking water does not increase the likelihood of adverse health effects. On the other hand, the values of HQ for children are a little bit above 1 (1.4), pointing out that they are a sensitive groups and that there is a need for further monitoring and analysis of this issue. The results of simulations of HQ values for private wells with 90% confidence show that the levels of HQ are well above 1 (2.12–5.76) for all the analyzed groups, indicating that drinking water from private wells could pose a high health risk. Nitrate concentrations were converted to the binary response of 0 or 1. In case that the nitrate concentration in the water sample was below 10 mg/L, the value of 0 was designated, and if the concentration was above 10 mg/L, the value of 1 was designated. The nitrate concentrations in the water samples of 10 mg/L were chosen based on the MCL for nitrate–N set by the U.S. Environmental Protection Agency (EPA) under the Safe Drinking Water Act [51]. Similar conversions were applied to livestock and cropping activities. The value of 1 was designated if wells were located near the livestock and cropping activities. Otherwise, the value of 0 was designated. The values of well depths and pH were measured.

As for Fe content, the considerably higher Fe content, above MCL (712.2 µg/L) was found in private wells (rural area). Aamodt et al. [23] found that the risk of developing IBD, including UC and CD, was associated with high iron content in drinking water. The relative risk of developing UBC increased by 21% when the iron content of drinking water increased by 0.1mg/L. Furthermore, they found an association between these diseases and aluminum in water, color, and turbidity. The author also suggests that there are two mechanisms for explaining this connection. High iron concentration acts as a catalyst for oxidative stress causing inflammation and/or increasing the rate of cell mutation, and second, iron content stimulates bacterial growth and increases the likelihood of an inappropriate immune response in genetically predisposed individuals. As a result of the study, Aamodt et al. [23] stressed the connection between the Fe compound in drinking water and IBD.

Arsenic (As) concentration above MCL (≤10 µg/L) was found in the rural water supply of Vukovar-Srijem County at three locations: Nijemci 45 µg/L; Markušica 213 µg/L; and Privlaka 83 µg/L. Patients were informed about this threat. As for the duration of water consumption, as well as the type of water supply and water quality, the result of our study is in agreement with Manber et al. [22] and Segerman et al. [8], who also reported that there is no significant correlation between the amount or source of water at the presence or absence of IBD.

## 5. Conclusions

It should be noted that this study contributes to new cognitions in clarifying IBD etiology. Moreover, it contributes to the awareness of healthcare professionals, as well as the patients, about the importance of environmental factors as the factors that reduce the risk of IBD occurrence. The findings of this study can be understood as a confirmation of how important drinking water quality for IBD is, even though the studied parameters did not show a significant impact on IBD phenotype or activity. Here, the limitation in cross-sectional studies must be taken into consideration, especially in collecting sufficient patients in a short period of time. High NO_3_^−^ (192 mg/L; private well in Požega-Slavonia County, 73.04 mg/L; rural water supply and private well in Virovitica-Podravina County, 62.1 mg/L in rural water supply, and 72.3 mg/L in a private well in Vukovar-Srijem County), Fe (712.2 µg/L; rural water supply, 223.5 µg/L; private well in Vukovar-Srijem County) and Mn (184.8 µg/L; rural water supply in Vukovar-Srijem County) that are above MCL are key components of future research that needs to be investigated. One special concern is high (213 µg/L) arsenic content in the rural water supply (Vukovar-Srijem County). Furthermore, special attention should be given to children as a sensitive group and to intensify the monitoring of nitrate leaching in rural areas with intensive agricultural production. Despite the advances in understanding the onset of IBD, it is necessary to change life habits and further investigate factors that may affect the natural course of the disease before the onset of clinical symptoms of the disease. It should be noted that the major concern is the lack of continuous monitoring of drinking water quality, especially in areas where contamination from diffuse, and point source pollution from agricultural production, often occurs. Furthermore, from a public health point of view, this study provides a good foundation for the establishment of the population registry of IBD for the eastern Croatia area.

## Figures and Tables

**Table 1:** The phenotypic characteristic of inflammatory bowel disease (IBD) patients.

Phenotypic Characteristic		Ulcerative Colitis (*N* = 198)	*N* (%)	Crohn’s Disease (*N* = 114)	*N* (%)	*p*
Disease duration	Age at diagnosis Medians (Q1, Q3)	41.0 (30,53)		35.5 (26,48)		0.009 *
	Disease duration Medians (Q1, Q3)	7.0 (5,12)		7.0 (4,12)		0.597 *
Montreal Classification	Age at diagnosis (A)		_	A1: (Age ≤ 16)	6 (5.0%)	_
			_	A2: (17–40)	59 (52.0%)	_
			_	A3: (>40)	49 (43.0%)	_
	Extent (E)/ Location (L)	E1 (Proctitis)	51 (26.0%)	L (Ileal)	21 (18.0%)	_
		E2 (Left side)	117 (59.0%)	L2 (Colonic)	61 (53.0%)	_
		E3 (extensive colitis)	30 (15.0%)	L3 (Ileocolonic)	32 (28.0%)	_
				L4 (Upper GI)	0	_
	Behavior	_		B1 (Inflammatory)	66 (58.0%)	
		_		B2 (Structuring)	39 (34.0%)	_
		_		B3 (Penetrating)	9 (8.0%)	
		_		p: (Perianal)	0	
Disease activity	Mayo index					0.335 ^†^
	Active Disease	79 (40.0%)		_		
	Inactive Disease	119 (60%)		_		
	HBI					
	Active Disease	_		39 (34.0%)		
	Inactive Disease	_		75 (66.0%)		

HBI, Harvey–Bradshaw index; median (Q1, Q3) interquartile range. * Mann Whitney U test, ^†^ Fisher’s exact test.

**Table 2:** The distribution of patients according to the county, length of the residence in county, and settlement in urban or rural areas.

	Crohn’s Diseases (%)	Ulcerative Colitis (%)	*p*
Distribution of patients according to the county			
Vukovar-Srijem	40 (35.81)	128 (64.65)	<0.001 *
Požega-Slavonia	34 (29.82)	40 (20.20)	
Virovitica-Podravina	40 (35.09)	30 (15.15)	
Total	114 (100)	198 (100)	
Distribution of patients according to the length (year) of the residence in county			
<9	1 (0.88)	6 (3.03)	0.312 ^±^
10–19	5 (4.39)	9 (4.55)	
20–29	24 (21.05)	28 (14.14)	
>30	84 (73.68)	155 (78.28)	
Total	114 (100)	198 (100)	
Distribution of patients according to the settlement in urban or rural areas			
Urban area	70 (61.40)	103 (52.02)	0.137 *
Rural area	44 (38.60)	95 (47.98)	
Total	114 (100)	198 (100)	

* x^2^ test, ^±^ Fishers’ exact test.

**Table 3:** Results of water physiochemical analysis according to the water source.

PSC	NO_3_^−^ (MCL < 50 mg/L)				Chloride (MCL 250 mg/L)				Fe (MCL 200 µg/L)				Mn (MCL 50 µg/L)				Al (MCL 200 µg/L)			
	Max	Min	Av	Stdev	Max	Min	Av	Stdev	Max	Min	Av	Stdev	Max	Min	Av	Stdev	Max	Min	Av	Stdev
PWS	8.87	<0.031	5.73	4.44	8.1	6.3	7.55	1.17	<50		-	-	<35		-	-	113.4	<35	-	-
PW	192	5.83	70.69	49.01	118.7	4.7	55.16	31.14	<50	66.7	-	-	<35		-	-	<35		-	-
	pH (6.5–9.5)				EC (2500 µS/cm)				Total hardness (°dH)				Color (20 Pt/Co)				Ar (MCL < 10 µg/L)			
PWS	7.9	7.4	7.6	0.22	424	74.4	310.1	159.5	21.58	3.28	15.33	7.8	35	0	9.75	16.94	-	-	-	-
PW	73.04	<0.1	7.8	35.05	57.18	0	26.4	28.8	43.54	17.32	22.6	7.3	51	0	14.8	16.4	-	-	-	-
VPC	NO_3_^−^ (MCL < 50 mg/L)				Chloride (MCL 250 mg/L)				Fe (MCL 200 µg/L)				Mn (MCL 50 µg/L)				Al (MCL 200 µg/L)			
PWS	21.69	0.1	3.6	7.3	20.7	0	10.9	7.3	31	0	10	13.8	26	2	12.1	7.5	240	0	84.4	86.5
RWS	73.04	0.1	7.8	35.05	57.18	0	26.4	28.8	5	<1	-	-	19	9	14.6	15.1	26	22	23.6	2.1
PW	73.04	3.98	37.9	19.8	172.98	13.26	61.4	62.6	40	0	12.5	14.9	33	8	21	11.6	730	0	121	248
	pH (6.5–9.5)				EC (2500 µS/cm)				Total hardness (°dH)				Color (20 Pt/Co)				Ar (MCL < 10 µg/L)			
PWS	6.9	6.4	6.8	0.23	526	69.4	320.1	168.5	21.25	4.39	16.7	7	37	0	9.84	17.03	-	-	-	-
RWS	54.9	0	5.3	38.5	325	65.24	120.6	99.8	35.6	21.5	18.3	7.4	42.5	0	10.2	17.1	-	-	-	-
PW	68.04	1	6.7	32.25	63.18	0	34.4	35.8	42.58	17.32	22.6	7.3	55	0	15.8	17.4	-	-	-	-
VSC	NO_3_^−^ (MCL < 50 mg/L)				Chloride (MCL 250 mg/L)				Fe (MCL 200 µg/L)				Mn (MCL 50 µg/L)				Al (MCL 200 µg/L)			
PWS	14.59	<0.6	7.7	5.4	37.3	9	23.6	10.2	60.7	0	8.7	22.9	45	0	29.7	16.2	92	0	42.2	28.2
RWS	62.1	<0.6	44.7	18.4	56.4	12.4	38.9	21.3	712.2	<50	-	-	184.8	35	154.4	42.9	<35		-	-
PW	72.3	0.73	22.7	31.3	55.8	12.4	16.9	11.2	223.5	<50	-	-	<35		-	-	<35		-	-
	pH (6.5–9.5)				EC (2500 µS/cm)				Total hardness (°dH)				Color (20 Pt/Co)				Ar (MCL < 10 µg/L)			
	Max	Min	Av	Stdev	Max	Min	Av	Stdev	Max	Min	Av	Stdev	Max	Min	Av	Stdev	Max	Min	Av	Stdev
PWS	7.68	7.2	7.6	0.2	705	394	482	92	24.63	5.8	17.8	9	7	0	1.8	1.9	-	-	-	-
RWS	7.6	7.3	7.4	0.1	1077	499	853	248	33.5	11.6	19.8	8.4	124	2	35.3	59.3	213	45	113.7	88.1
PW	8.1	7.3	7.6	0.3	894	398	699	164	44.54	18.56	23.5	7.9	33	0	10.7	14.2	-	-	-	-

Stdev = standard deviation; PSC = Požega-Slavonia County; VPC = Virovitica-Podravina; VSC = Vukovar-Srijem; PWS = Public Water Supply; RWS = Rural Water Supply; PW = Private well.

**Table 4:** The impact of type of water supply and the water quality on IBD phenotype.

	Crohn’s Diseases (%)	Ulcerative Colitis (%)	*p*
The patient’s distribution according to type of water supply			
Public water supply	82 (71.93)	147 (74.25)	0.111 ^±^
Rural water supply	22 (19.30)	36 (18.18)	
Well	10 (8.77)	15 (7.58)	
Total	114 (100)	198 (100)	
The patient’s distribution according to water quality			
Below the limit	23 (20.18)	31 (15.66)	0.389 *
Over the limit	91 (79.82)	167 (84.34)	
Total	114 (100)	198 (100)	

* x^2^ test, ^±^ Fisher’s exact test.

**Table 5:** The type of drinking water supply as well as the water quality impact on IBD activity.

	Crohn’s Diseases (%)	Ulcerative Colitis (%)	*p*
The patient’s distribution according to the type of water supply			
Public water supply	141 (72.68)	88 (74.58)	0.277 ^±^
Rural water supply	37 (19.07)	21 (17.80)	
Well	16 (8.25)	9 (7.63)	
Total	194 (100)	118 (100)	
The patient’s distribution according to the water quality			
Below the limit	35 (18.04)	19 (16.10)	0.776 *
Over the limit	159 (81.96)	99 (83.90)	
Total	194 (100)	118 (100)	

* x^2^ test, ^±^ Fisher’s exact test.

**Table 6:** Values of (and hazard quotient (HQ) values for) public water and groundwater well supply.

Parameter	Children	Female	Male
Public water supply			
Mean	0.71	0.25	0.29
Standard deviation	3.02	0.88	1
P90	1.4	0.52	0.6
Groundwater wells			
Mean	3.3	1.2	1.35
Standard deviation	2.77	0.98	1.15
P90	5.76	2.12	2.31

**Table 7:** Results of logistic regression.

Variables	Wald Statistic (z Statistic)	*p*
Depth of Well	0.074	0.941
pH	−0.600	0.548
Crop production	−0.894	0.371
Livestock	0.005	0.996

## References

[1] Misra S.M. (2014). Integrative therapies and pediatric inflammatory bowel disease: The current evidence. Children.

[2] Fofanova T.Y., Petrosino J.F., Kellermayer R. (2016). Microbiome-epigenome interactions and the environmental origins of inflammatory bowel diseases. J. Pediatr. Gastroenterol. Nutr..

[3] Holik D., Včev A., Milostić-Srb A., Salinger Ž., Ivanišević Z., Včev I., Miškulin M. (2019). The effect of daily physical activity on the activity of inflammatory bowel diseases in therapy-free patients. Acta Clin. Croat..

[4] Burisch J. (2014). Crohn’s disease and ulcerative colitis. Occurrence, course and prognosis during the first year of disease in a European population-based inception cohort. Dan. Med. J..

[5] Vegh Z., Kurti Z., Lakatos P. (2017). The epidemiology of inflammatory bowel diseases from west to east. J. Dig. Dis..

[6] Lofthus E.V. (2004). Clinical epidemiology of inflammatory bowel disease: Incidence, prevalence, and environmental influences. Gastroenterology.

[7] Molodecky N.A., Soon I.S., Rabi D.M., Ghali W.A., Ferris M., Chernoff G., Benchimol E.I., Panaccione R., Ghosh S., Barkema H.W. (2012). Increasing incidence and prevalence of the inflammatory bowel diseases with time, based on systematic review. Gastroenterology.

[8] Segerman F., Clarkson S., Sjöberg K. (2019). Marked regional variations in the prevalence of inflammatory bowel disease in a limited geographical region are not associated with compounds in the drinking water. Scand. J. Gastroenterol..

[9] Yang S.K., Loftus E.V., Sandborn W.J. (2001). Epidemiology of inflammatory bowel disease in Asia. Inflamm. Bowel Dis..

[10] Thia K.T., Loftus E.V., Sandborn W.J., Yang S.K. (2008). An update on the epidemiology of inflammatory bowel disease in Asia. Am. J. Gastroenterol..

[11] Burisch J., Pederson N., Cukovic-Cavka S., Turk N., Kaimakliotis I., Duricova D., Bortlik M., Shonová O., Vind I., Avnstrøm S. (2014). Environmentlal factors in a population-based inception cohort of IBD patients in Europe-an ECCO-Epi Com study. J. Crohns Colitis.

[12] Ng S.C., Bernstein C.N., Vatn M., Lakatos P.L., Loftus E.V., Tysk C., O’Morain C., Moum B., Colombel J.F. (2013). Geographical variability and environmental risk factors in inflammatory bowel disease. Gut.

[13] Pezerović D., Klarin I., Zulj M., Majnarić L.J., Khaznadar E., Vcev A. (2014). Incidence and prevalence of inflammatory bowel disease in Vukovarsko-Srijemska County, Croatia, 1991–2000 and 2001–2010: A population-based study. Coll. Antropol..

[14] Klarin I., Majnarić L.J., Jovanović Ž., Nakić D., Včev I., Včev A. (2013). Epidemiology and clinical presentation of inflammatory bowel disease in Zadar County, Croatia. Coll. Antropol..

[15] Moum B., Hovde R., Hrivik M.L. (2014). What have we learnt about the role of the environment and natural course of IBD in the new millennium? 20-year follow-up of the IBSEN cohort. Dig. Dis..

[16] Burisch J., Munkholm P. (2015). The epidemiology of inflammatory bowel disease. Scand. J. Gastroenterol..

[17] Aujnarain A., Mack D.R., Benchimol E.I. (2013). The role of the environment in the development of pediatric inflammatory bowel disease. Curr. Gastroenterol. Rep..

[18] Soon I.S., Molodecky N.A., Rabi D.M., Ghali W.A., Barkema H.W., Kaplan G.G. (2012). The relationship between urban environment and the inflammatory bowel diseases: A systematic review and meta-analysis. BMC Gastroenterol..

[19] Morgan X.C., Tickle T.L., Sokol H., Gevers D., Devaney K.L., Ward D.V., Reyes J.A., Shah S.A., Leleiko N., Snapper S.B. (2012). Dysfunction of the intestinal microbiome in inflammatory bowel disease and treatment. Genome Biol..

[20] Vcev A., Pezerovic D., Jovanovic Z., Nakic D., Vcev I., Majnaric L. (2015). A retrospective, case-control study on traditional environmental risk factors in inflammatory bowel disease in Vukovar-Srijem County, North-Eastern Croatia, 2010. Wien. Klin. Wochenschr..

[21] Rogler G., Zeitz J., Biedermann L. (2016). The search for causative environmental factors in inflammatory bowel disease. Dig. Dis..

[22] Manbeer S., Singh P., Tadepalli S., Nookala V. (2018). The association of water and inflammatory bowel disease. Am. J. Gastroenterol..

[23] Aamodt G., Bukholm G., Jahnsen J., Moum B., Vatn M.H. (2008). The association between water supply and inflammatory bowel disease based on a 1990–1993 cohort study in Southeastern Norway. Am. J. Epidemiol..

[24] Fathmawati F., Fachiroh J., Sutomo A.H., Putra D.P.E. (2018). Origin and distribution of nitrate in water well of settlement areas in Yogyakarta, Indonesia. Environ. Monit. Assess..

[25] World Health Organization (2004). Water, Sanitation and Health Team. Guidelines for Drinking-Water Quality. Vol. 1, Recommendations.

[26] Izvještaj o Zdravstvenoj Ispravnosti Vode za Ljudsku Potrošnju u Republici Hrvatskoj. https://www.hzjz.hr/sluzba-zdravstvena-ekologija/izvjestaj-o-zdravstvenoj-ispravnosti-vode-za-ljudsku-potrosnju-u-republici-hrvatskoj-za-2018-godinu/.

[27] The Environmental Implementation Review 2019. Country Report Croatia. https://ec.europa.eu/environment/eir/pdf/report_hr_en.pdf.

[28] Report from the Commission to the Council and the European Parliament. https://ec.europa.eu/environment/water/waternitrates/pdf/nitrates_directive_implementation_report.pdf.

[29] Brkić Ž., Kuhta M., Larva O., Gottstein S. (2019). Groundwater and connected ecosystems: An overview of groundwater body status assessment in Croatia. Environ. Sci Eur..

[30] Okoliš na Dlanu I-2018. http://www.haop.hr/sites/default/files/uploads/publications/2018-10/Okolis%20na%20dlanu%20I%20%20-%202018.pdf.

[31] EUROSTAT Agri-Environmental Indicator—Mineral Fertiliser Consumption. https://appsso.eurostat.ec.europa.eu/nui/show.do?dataset=aei_fm_usefert&lang=en.

[32] Croatian Bureau of Statistics, CBS Statistical Yearbook of the Republic of Croatia. https://www.dzs.hr/Hrv_Eng/ljetopis/2018/sljh2018.pdf.

[33] Kogevinas M., Chatzi L. (2015). Cross-Sectional Studies.

[34] Soleimani H., Nasri O., Ojaghi B., Pasalari H., Hosseini M., Hashemzadeh B., Kavosi A., Masoumi S., Radfard M., Adibzadeh A. (2018). Data on drinking water quality using water quality index (WQI) and assessment of groundwater quality for irrigation purposes in Qorveh & Dehgolan, Kurdistan, Iran. Data Brief..

[35] Shalyari N., Alinejad A., Hashemi A.H.G., Radfard M., Dehghani M. (2019). Health risk assessment of nitrate in groundwater resources of Iranshahr using Monte Carlo simulation and geographic information system (GIS). MethodsX.

[36] World Health Organization. Nutrients in Drinking Water.

[37] Akbari H., Soleimani H., Radfard M., Abasnia A., Hashemzadeh B., Akbari H., Adibzadeh A. (2018). Data on investigating the nitrate concentration levels and quality of bottled water in Torbat-e Heydarieh, Khorasan razavi province, Iran. Data Brief.

[38] USEPA Integrated risk information system United States Environmental Protection Agency. https://tools.niehs.nih.gov/srp/1/Resources/arzuaga_iris_20091019.pdf.

[39] Chen J., Wu H., Qian H., Gao Y. (2017). Assessing nitrate and fluoride contaminants in drinking water and their health risk of rural residents living in a semiarid region of Northwest China. Expo. Health.

[40] Liu A., Ming J., Ankumah R.O. (2005). Nitrate contamination in private wells in rural Alabama, United States. Sci. Total Environ..

[41] James G., Witten D., Hastie T., Tibshirani R. (2013). An Introduction to Statistical Learning with applications in R.

[42] AOD: Analysis of overdispersed data. R Package Version 1.3.1. https://cran.r-project.org/web/packages/aod/aod.pdf.

[43] Probabilistic Modelling for Assessment of Exposure via Drinking Water. Final Report of Project Defra WT1263/DWI 70/2/273. http://dwi.defra.gov.uk/research/completed-research/reports/DWI70-2-273.pdf.

[44] Cholapranee A., Ananthakrishnan A.N. (2016). Environmental hygiene and risk of inflammatory bowel diseases: A systematic review and meta-analysis. Inflamm. Bowel Dis..

[45] Package ‘Fitdistrplus’ Help to Fit of a Parametric Distribution to Non-Censored or Censored Data. https://rdrr.io/cran/fitdistrplus/.

[46] Dutta A.M., Chacko A. (2016). Influence of environmental factors on the onset and course of inflammatory bowel disease. World J. Gastroenterol..

[47] Lakatos P.L. (2009). Environmental factors affecting inflammatory bowel disease: Have we made progress?. Dig. Dis..

[48] López-Serrano P., Pérez-Calle J.L., Pérez-Fernández M.T., Fernández-Font J.M., Boixeda de Miguel D., Fernández-Rodríguez C.M. (2010). Environmental risk factors in inflammatory bowel diseases. Investigating the hygiene hypothesis: A Spanish case-control study. Scand. J. Gastroenterol..

[49] Sood A., Amre D., Midha V., Sharma S., Sood N., Thara A., Bansal M., Juyal G., Thelma K.B., Seidman E. (2014). Low hygiene and exposure to infections may be associated with increased risk for ulcerative colitis in a North Indian population. Ann. Gastroenterol..

[50] Wang M.H., Achkar J.P. (2015). Gene-environment interactions in inflammatory bowel disease pathogenesis. Curr. Opin. Gastroenterol..

[51] (1996). Environmental Indicators of Water Quality in the United States. EPA. https://nepis.epa.gov/Exe/tiff2png.cgi/2000CZIX.PNG?-r+75+-g+7+D%3A%5CZYFILES%5CINDEX%20DATA%5C95THRU99%5CTIFF%5C00000522%5C2000CZIX.TIF.

